# Notes on Preparations for the Sick

**Published:** 1910-06

**Authors:** 


### Notes on Preparations for the Sick.

**Brussels International Exhibition.**—Medical men who visit the Brussels Exhibition will be interested in inspecting the handsome exhibit of Burroughs, Wellcome & Co., which occupies a commanding position near the entrance to the Galerie de l'Industrie Britannique. Their comprehensive display includes a wide range of "Tabloid" Products, Medical Equipments, Hypodermic Syringes and Cases, Compressed Dressings, "Wellcome" Brand Chemicals, "Kepler" and "Hazeline" Preparations, "Ernutin," "Hemisine," "Soloid," "Vaporole" and "Enule" products, "Tabloid" Photographic Chemicals, etc.

Although pioneers in the pharmacy of the thyroid gland, this firm has taken yet another step forward by standardising "Tabloid" Thyroid Gland to contain a definite proportion of iodine in organic combination.

"Orsudan" and "Soamin," the new arylarsonates, are on view. These have been used with success in the treatment of syphilis, malaria and other protozoal diseases.

An entirely new therapeutic agent shown is "Lodal," an oxidation product of laudanosine (an alkaloid occurring in opium), which was first synthesised and investigated by research workers in the laboratories of the "Wellcome" Chemical Works.

Pituitary (Infundibular) Gland, a medicinal animal substance which is attracting attention in modern therapeutics, is exhibited in the form of "Vaporole" and "Tabloid" products.

A striking example of advance in pharmacy is illustrated by the fact that, being unable to obtain medicinal herbs conforming to their high standard of efficiency, this firm now grows its own on a large scale.

In addition to the pharmaceutical exhibits, there is displayed a unique collection of historical "Tabloid" Medical Cases carried by famous explorers and expeditions.

---

**Hydropyrin.**—ROBORAT COMPANY, London, E.C.—A sample of this compound Lithium Salicylic Salt has been sent to us. It is a soluble powder, crystalline, with no disagreeable taste. It contains :—

| | |
|---|---|
| Acetyl-salicylic Acid .. .. | 96.24 per cent. |
| With Lithium .. .. .. | 3.76 per cent. |

In half-gramme doses it should be an efficient remedy, and its solubility gives it a distinct advantage over many of the salicylic compounds. It is obviously a salicylic drug, but the lithium element may prove to be an advantage in cases of gout.

On the whole, the properties of Hydropyrin correspond with those of Aspirin, but it has the advantage of being soluble and not irritating the stomach, for which reasons patients can tolerate it more easily. Hydropyrin—like the salicyl-preparations generally—is a specific anti-rheumatic remedy, in addition it is a reliable antipyretic free from any by-effects, and in certain cases it acts well as an anti-neuralgic. Of no minor importance is the diaphoretic and diuretic action, which Hydropyrin shows in a marked degree. The average dose is 7½—15 grains, while as a maximum dose 45 grains given in twenty-four hours might be considered.

---

**Meat Port.**—BENDLE LIMITED, London, S.E.—A nutritive meat wine, prepared from old tawny port, and containing meat nutrient which is estimated to be about 7 per cent.

A wineglassful contains 3½ fluid drachms of alcohol, and about 22 grains of meat nitrogenous extract, with 70 grains of glucose.

It is very palatable, and should be of great value in cases of debility when an alcoholic nutrient is desired.

---

**Iodine Vasogen.**—E. J. Reed & Co., London.—The ordinary preparations of Iodine do not lend themselves readily to absorption, they stain the skin, and at the same time harden it.

The combination with Vasogen has been found by repeated trials and chemical tests to be more rapidly absorbed than any other compound containing Iodine, whilst it may be used for an indefinite period without causing discolouration of the skin.

From the clinical experiments of Dr. Dahmen and others it has been proved that Iodine may be traced in the urine within three and a half hours after inunction with Iodine Vasogen, and that traces may be found for a whole day after.

Iodine Vasogen is prepared in two strengths, 6 per cent. and 10 per cent., the former being generally preferred for superficial induration, chronic neuritis, parasitic skin diseases, etc., and the latter for deep-seated chronic swellings, glandular enlargements and joint affections.

---

**"Milk Stout."**—Ashton Gate Brewery Co. Ltd., Bristol.—This is a beverage which contains the carbohydrate (lactose) present in milk, and is specially recommended for invalids. We have examined a sample, and find it to be as represented, a pleasant and mildly alcoholic drink.

The alcohol was estimated, and found to the extent of 4 per cent. approximately. It can thus be taken by many persons who would find the use of stronger alcoholic preparations undesirable, and it should be a decidedly nourishing and useful beverage.

---

**"Roncegno"** (a natural arsenical iron mineral water from Mount Tesobo, Austria).—Importer: Richard Davis, 20 Maddox Street, Regent Street, London, W.—This is a naturally occurring "tonic water," and is recommended to be taken by adults in doses of one to four tablespoonfuls.

It is faintly yellow in colour, and possesses an astringent taste.

We have chemically examined a specimen, and have identified iron in the ferric state with traces of arsenic.

